# PACER lncRNA regulates COX-2 expression in lung cancer cells

**DOI:** 10.18632/oncotarget.28190

**Published:** 2022-02-04

**Authors:** Samuel Z. Desind, Joseph R. Iacona, Christina Y. Yu, Antonina Mitrofanova, Carol S. Lutz

**Affiliations:** ^1^Department of Microbiology, Biochemistry, and Molecular Genetics, Rutgers Biomedical and Health Sciences, New Jersey Medical School, School of Graduate Studies, Newark, NJ 07103, USA; ^2^Department of Health Informatics, Rutgers School of Health Professions, Rutgers Biomedical and Health Sciences, Newark, NJ 07107, USA; ^3^Rutgers Cancer Institute of New Jersey, Rutgers, The State University of New Jersey, New Brunswick, NJ 08901, USA; ^*^These authors contributed equally to this work

**Keywords:** PACER, COX-2, PGE_2_, lung adenocarcinoma, inflammation

## Abstract

Long noncoding RNAs (lncRNAs) are known to regulate gene expression; however, in many cases, the mechanism of this regulation is unknown. One novel lncRNA relevant to inflammation and arachidonic acid (AA) metabolism is the p50-associated COX-2 extragenic RNA (PACER). We focused our research on the regulation of PACER in lung cancer. While the function of PACER is not entirely understood, PACER is known to play a role in inflammation-associated conditions. Our data suggest that PACER is critically involved in COX-2 transcription and dysregulation in lung cancer cells.

Our analysis of The Cancer Genome Atlas (TCGA) expression data revealed that PACER expression is significantly higher in lung adenocarcinomas than normal lung tissues. Additionally, we discovered that elevated PACER expression strongly correlates with COX-2 expression in lung adenocarcinoma patients. Specific siRNA-mediated knockdown of PACER decreases COX-2 expression indicating a direct relationship. Additionally, we show that PACER expression is induced upon treatment with proinflammatory cytokines to mimic inflammation. Treatment with prostaglandin E2 (PGE_2_) induces both PACER and COX-2 expression, suggesting a PGE_2_-mediated feedback loop. Inhibition of COX-2 with celecoxib decreased PACER expression, confirming this self-regulatory process. Significant overlap between the COX-2 promotor and the PACER promotor led us to investigate their transcriptional regulatory mechanisms. Treatment with pharmacologic inhibitors of NF-κB or AP-1 showed a modest effect on both PACER and COX-2 expression but did not eliminate expression. These data suggest that the regulation of expression of both PACER and COX-2 is complex and intricately linked.

## INTRODUCTION

Lung cancer remains the deadliest cancer in the United States, causing approximately 135,720 deaths in 2020 [1]. Non-small cell lung cancer (NSCLC) is responsible for 85% of all lung cancer cases in the United States [1]. NSCLC includes several subtypes: large cell carcinoma, squamous cell carcinoma, and adenocarcinoma. Lung adenocarcinoma (LUAD) and squamous cell carcinoma (LUSC) are the two major subtypes. LUAD is derived from glandular cells of the lungs, while LUSC develops from epithelial tissue [2, 3]. While the phenotypic and mutational characteristics of LUAD and LUSC differ, both exhibit a high rate of treatment-resistant mutations, frequently leading to the failure of traditional targeted chemotherapeutics [4–8].

The arachidonic acid (AA) pathway is a major inflammatory pathway dysregulated in lung cancer. The products of the AA pathway are a group of clinically important molecules known as eicosanoids. Cyclooxygenase 2 (COX-2) is a main synthase of the AA pathway. COX-2, a key regulator of the inflammatory response, is significantly overexpressed in many types of cancer, including breast, ovarian, colorectal, and lung cancers [9–13]. COX-2 converts AA into prostaglandin H2 (PGH_2_). PGH_2_ is subsequently converted by microsomal prostaglandin E synthase 1 (mPGES-1) into prostaglandin E2 (PGE_2_). PGE_2_ is a terminal product of the AA pathway [14]. PGE_2_ acts through one of four G protein-coupled prostaglandin E2 receptors activating cAMP/PKA-mediated regulation of several pathways, including the MAPK/Erk, PI3K, ATK, and β-catenin pathways [15–17]. The normal physiological roles of PGE_2_ include regulating pain, kidney function, apoptosis, cell clearance, cell growth, immune cell regulation, and inflammation [18, 19].

In the context of cancer, PGE_2_ acts as both a proinflammatory and immunosuppressive molecule. Many cancers, including lung cancer, overexpress COX-2, leading to increased PGE_2_ production within the tumor microenvironment (TME) [20]. PGE_2_ also alters immune cell polarization in the TME, creating pro-tumorigenic conditions [19, 21, 22]. Increased levels of PGE_2_ within the TME of NSCLC have been shown to inhibit CD8^+^ T-cell, T_H_1, and NK cell activation, stop dendritic cell maturation and encourage the recruitment of regulatory-T cells and T_H_2 cells [23, 24]. Additionally, increased PGE_2_ can directly increase cancer cell proliferation, invasion, angiogenesis, and survival [17, 18, 25–28].

Despite the significant efforts made to decipher the mechanisms of COX-2 transcription, many unanswered questions remain regarding how changes in COX-2 expression affect phenotypic changes in tumor growth and cancer progression. We have previously shown that COX-2 expression is regulated by alternative polyadenylation and miRNA regulation; however, we appreciate that COX-2 regulation can occur on many levels [29–35].

Several noncoding RNAs, including long noncoding RNAs (lncRNAs), are now known to modulate transcription and post-transcriptional regulation of crucial inflammatory genes with roles in lung cancer tumorigenesis [36]. Noncoding RNA makes up a majority of the human genome [37]. LncRNAs are defined as RNAs that are 200 nucleotides or greater in length and do not undergo translation into functional proteins. LncRNAs serve many functional and structural roles that vary significantly throughout the genome [38]. LncRNAs have been shown to regulate chromatin modification and mRNA transcription, stability and turnover in a tissue or cell type-specific manner [37].

Rapicavoli et al. characterized the COX-2 divergent (Ptgs2os) noncoding gene that is present antisense to the COX-2 gene on chromosome 1 in *Mus musculus*. They found that Ptgs2os expression was induced by several immune stimulatory factors, including TNFα, IL-1β, and agonists of the TLR4 receptor [39]. At this time, a gene homologous to Ptgs2os had not been identified in humans. While previous investigations identified a noncoding RNA (ncRNA) transcribed from the promoter and surrounding region of the human COX-2 locus [40], Krawczyk and colleagues first reported and initially characterized the Ptgs2os homologous lncRNA p50-associated COX-2 extragenic RNA known as PACER [41]. PACER, positioned on chromosome 1, lies upstream and antisense to the promotor region of the COX-2 gene. These researchers distinguished the role of PACER as a critical regulator of COX-2 transcription. They discovered regions of high RNA polymerase II (RNAP II) binding upstream of the COX-2 transcriptional start sequence using chromatin immunoprecipitation.

Furthermore, Krawczyk et al. identified CTCF/cohesin binding domains flanking the COX-2 locus. CTCF and cohesion alter the structure and accessibility of transcription factors to DNA. In the context of PACER and COX-2, CTCF/cohesin binding is thought to regulate chromatin structure and stability, allowing RNAP II to access the PACER and COX-2 locus [41]. Krawczyk et al. further described PACER’s capacity to regulate COX-2 mRNA through interaction with the p50 subunit of NF-κB. The p50 subunit, which lacks an activation domain, is constitutively bound to the promoter region of COX-2 and acts to inhibit transcription. Upon lipopolysaccharide stimulation to elicit an immune response, PACER transcription is activated. PACER RNA itself can bind to and effectively sequester the p50 subunit of NF-κB. Sequestration of p50 allows the p50-p65 heterodimer to bind to the COX-2 promoter and activate transcription [41]. Krawczyk et al. conclude by discussing the differing roles of COX-2 in early and late-stage cancer. They hypothesized that modulation of PACER expression may have significant clinical and therapeutic potential as a means of regulating the AA/COX-2/ PGE_2_ axis and altering immune cell polarization in the TME.

Another study by Sun et al. found a strong correlation between PACER and COX-2 expression in colorectal cancer (CRC) [42]. They showed siRNA knockdown of PACER expression in CRC cells led to a significant reduction in COX-2 expression. They also found that PACER promoted the proliferation, invasion, and metastasis of CRC cells and demonstrated the ability of PACER to regulate COX-2 transcription through NF-κB signaling. Sun et al. highlighted PACER as a potential therapeutic target for the regulation of COX-2 in CRC.

Recent investigations into PACER’s role as an inflammatory regulator and our expertise in AA signaling have prompted our investigations into how PACER regulates COX-2 expression in lung cancer. This study identifies a strong correlation between PACER and COX-2 expression in lung adenocarcinoma patient tissue samples using bioinformatic tools. Additionally, our siRNA-mediated knockdown of PACER demonstrates a direct relationship between PACER and COX-2 expression in lung cancer cells. We demonstrate that cytokine stimulation significantly increases both PACER and COX-2 expression in LUAD cell lines. We provide evidence of potential regulatory feedback mechanisms involving PGE_2_. Furthermore, we identify the possibility of additional mechanisms regulating PACER transcription by interrogating the COX-2 transcription factors AP-1 and NF-κB. We conclude that the regulatory mechanisms governing COX-2 and PACER expression are both complex and intricately linked.

## RESULTS

### PACER expression is increased in LUAD and correlates with COX-2 expression

**Figure 1:**
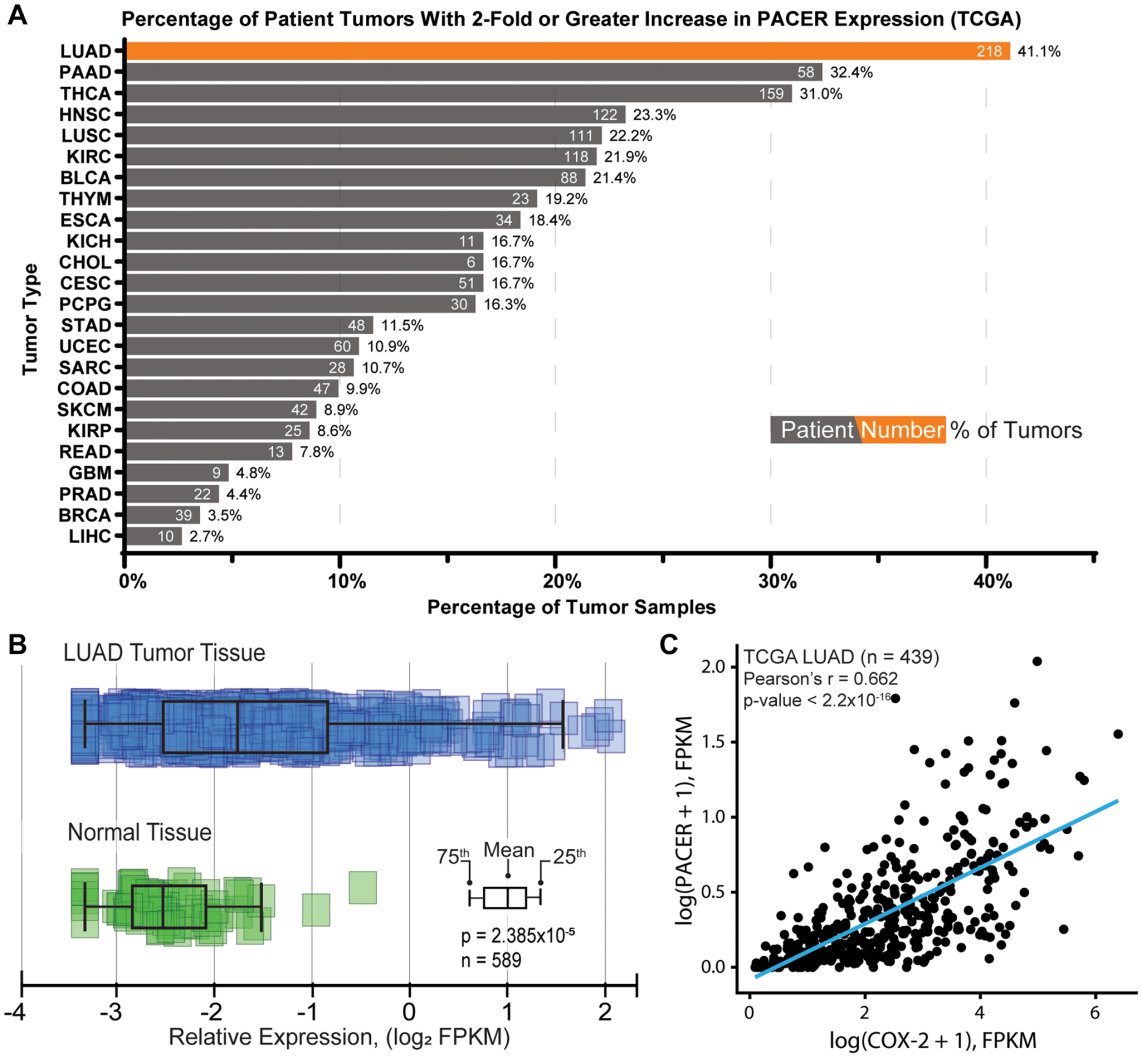
Characterization of PACER expression in lung adenocarcinoma (LUAD) and normal tissue. (**A**) Analysis of PACER expression across TCGA patient study cohorts by cancer type (ArrayStudio, QIAGEN). This analysis shows the percentage of patient tumor samples with a 2-fold or greater increase in PACER expression relative to normal tissue expression (number of patients with increased PACER expression shown in white). (**B**) Comparison of PACER FPKM values in normal lung tissue and LUAD patient tissues accessed from The Cancer Genome Atlas. PACER expression in LUAD tumor tissue is significantly different from normal tissue (*p* = 2.38 × 10^–5^). (**C**) Pearson correlation analysis comparing PACER and COX-2 relative expression values (FPKM) from LUAD patient tissues. PACER and COX-2 are significantly correlated in LUAD patient tissues (r = 0.662, *p* < 2.2 × 10^–16^).

We first analyzed the expression of PACER in patient cohorts for many of the cancer types available in TCGA. For each cancer type, we determined the number of patients with a 2-fold or greater increase in PACER expression relative to PACER expression in the corresponding normal tissue samples. We found that of the 24 cancer types analyzed, LUAD exhibited the highest percentage of patients with at least a 2-fold increase in PACER expression (41.1%) relative to normal lung tissue expression (Figure 1A). The significant number of LUAD patients exhibiting overexpression of PACER suggests that PACER dysregulation is a common marker of LUAD.

To examine the clinical relevance of PACER expression further, we compared the prevalence of PACER expression in normal lung tissues to expression in LUAD patient tumor tissues. We compared expression data from normal tissue and LUAD tumor tissue samples available in TCGA and found that PACER expression is significantly higher in LUAD patient tissues compared with normal lung tissues (*n* = 589, *p* = 2.38 × 10^−5^) (Figure 1B).

We continued to investigate the clinical role of PACER in LUAD by examining the relationship between bulk PACER and COX-2 expression in patient tumors. We performed correlation analysis on FPKM-normalized RNA-seq data from the TCGA LUAD cohort (*n* = 439, obtained through the Genomic Data Commons RNASeq Tool [43]) and found a significant positive correlation between PACER and COX-2 expression (*n* = 439, Pearson’s r = 0.662, *p* < 2.2 × 10^−16^) (Figure 1C). Notably, in this dataset, COX-2 was the gene most highly correlated with PACER expression levels. The correlation between PACER and COX-2 expression suggests that there is a strong regulatory relationship between PACER and COX-2 in LUAD tumors.

**Figure 2:**
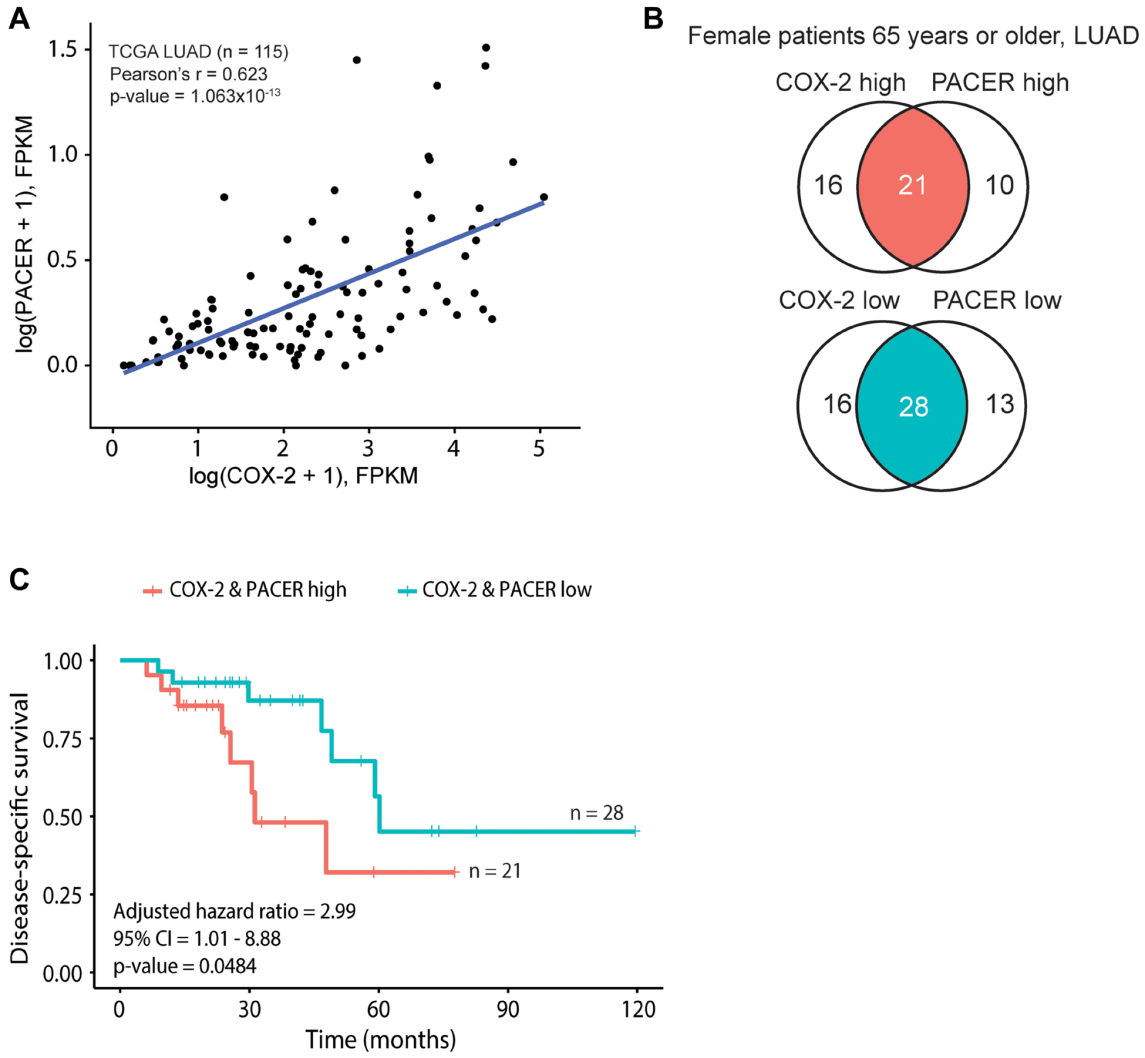
COX-2 and PACER coordinate expression contribute to significant survival differences in a subset of older female patients with lung adenocarcinoma (LUAD). Panels A-C show analyses conducted on The Cancer Genome Atlas (TCGA) lung LUAD datasets. (**A**) Scatterplot of COX-2 and PACER expression, with a fitted linear regression line. The Pearson’s correlation coefficient (r) and corresponding *p*-value calculated from the logged data are shown. (**B**) Venn diagrams depicting the identification of “COX-2 & PACER high” and “COX-2 & PACER low” groups in female patients aged 65 years or older. (**C**) Kaplan-Meier survival curves between the high and low patient groups defined by B, shown with the corresponding hazard ratio, confidence interval (CI), and Wald *p*-value calculated from a Cox proportional hazards model adjusted for tumor stage.

This link between PACER and COX-2 expression led us to investigate the effects of PACER and COX-2 coordinate expression on disease-specific survival (DSS) in the TCGA LUAD cohort. We matched samples with FPKM-normalized expression data from TCGA to corresponding clinical data accessed through cBioPortal [44]. We analyzed patient survival based on the tertials of PACER and COX-2 gene expression, comparing the top third of PACER and COX-2 expressing patients (COX-2 & PACER high) with the bottom third (COX-2 & PACER low). These two groups were then subjected to stratified KM survival analysis, where groups were further subset by gender, age, and clinical stage. Our analysis identified that female LUAD patients 65 years of age or older with high PACER and COX-2 expression had decreased DSS, with a Cox proportional hazards ratio of 2.99 (95% confidence interval (CI): CI [1.01–8.88]; *p*-value = 0.0484), adjusted for the clinical stage (Figure 2A–2C). The effects of PACER and COX-2 expression on DSS seems to be limited to specific patient subsets; however, our analysis relies on TCGA data and therefore provides a limited snapshot of patient and treatment conditions.

To evaluate if the relationship between COX-2 and PACER expression is LUAD specific, we performed both correlation and DSS analysis, as described above, in the TCGA LUSC patient cohort (*n* = 484). Correlation analysis between PACER and COX-2 expression in LUSC patients demonstrated lower association compared with the TCGA LUAD patient cohort (Pearson r = 0.597, *p* < 2.2 × 10^−16^) (data not shown). Notably, the coordinate expression of PACER and COX-2 did not affect DSS in female patients aged 65 or older (established in LUAD), with a clinical stage adjusted Cox proportional hazards ratio of 0.988 (95% CI [0.192–5.08]; *p*-value = 0.988) (Supplementary Figure 1A–1C).

Our results link the coordinate expression of PACER and COX-2 to the clinical outcomes (DSS), especially in female LUAD patients aged 65 and older. Additionally, we identified a difference in PACER and COX-2 coordinate expression between the lung cancer subtypes LUAD and LUSC. How PACER regulation of COX-2 differs between these morphologically distinct subtypes requires further investigation. However, our results indicate that cooperation may impact clinical outcomes and should be considered in developing strategies targeting this therapeutic axis.

### LUAD cell lines overexpress PACER

**Figure 3:**
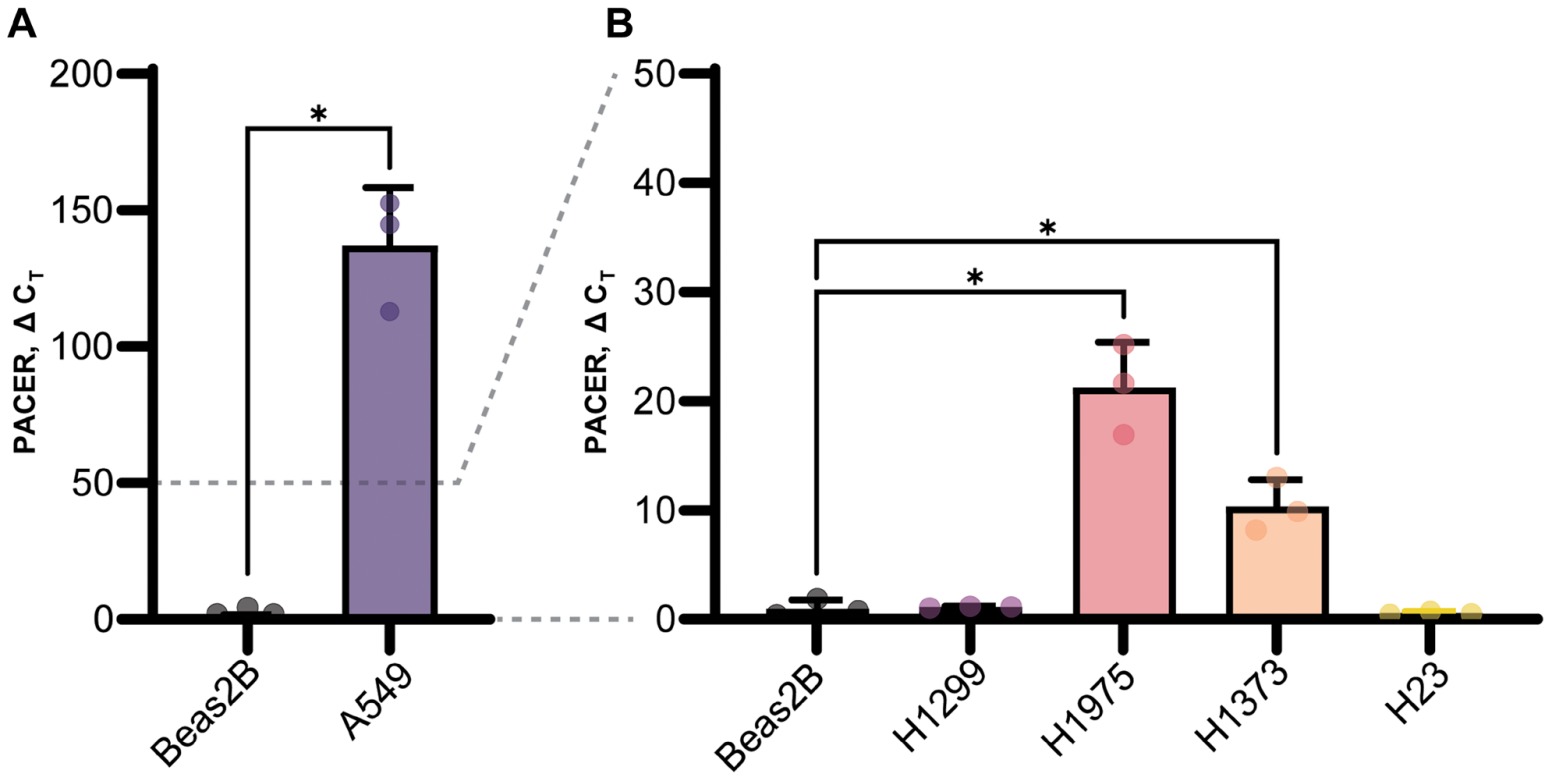
Baseline PACER expression in lung cell lines. PACER expression in several lung cancer cell lines (A549, H1299, H1975, H1373, H23) compared with PACER expression in the normal lung cell line Beas2B using real-time quantitative PCR (qRT-PCR). Raw CT values are normalized to the GAPDH expression in each cell line. (**A**) The A549 lung adenocarcinoma cell line expresses significantly higher levels of PACER compared with Beas2B cells (*p* = 0.008) and had higher expression that any other lung cancer cell line. (**B**) H1975 cells and the mutant KRAS adenocarcinoma cell line H1373 both exhibited increased PACER expression (*p* = 0.012, *p* = 0.015) compared with Beas2B cells. Error bars indicate standard error of the mean. ^*^ Indicates *p* < 0.05.

Establishing a cell-based system would allow us to further investigate the regulatory mechanisms of PACER and COX-2 expression. Using qRT-PCR, we measured baseline PACER expression in several lung cancer cell lines (A549, H1299, H1975, H1373, H23) (Figure 3A and 3B). We compared relative PACER expression in each lung cancer cell line to PACER expression in the normal lung tissue cell line Beas2B by performing qRT-PCR on RNA isolated from these cells. Raw cycle threshold (C_T_) values were first normalized to the GAPDH expression of each lung cancer cell line before comparing expression to Beas2B.

We found that PACER expression varied widely between LUAD cell lines. PACER was significantly upregulated in A549 cells (t(2.00) = 11.10, *p* = 0.008) (Figure 3A). PACER expression was also increased, but to a lesser degree, in H1975 cells (t(2.14) = 8.33, *p* = 0.012) and the mutant KRAS adenocarcinoma cell line H1373 (t(2.39) = 6.332, *p* = 0.015) (Figure 3B). High expression of PACER in A549 cells correlates with our previous observations of high COX-2 expression in this cell line [30]. These data suggest a strong regulatory link between PACER and COX-2 expression in A549 cells. We chose to conduct further experiments using A549 cells because of this observed correlation between PACER and COX-2 expression, as well as the constitutively high levels of both genes relative to expression in Beas2B.

### SiRNA knockdown of PACER

**Figure 4:**
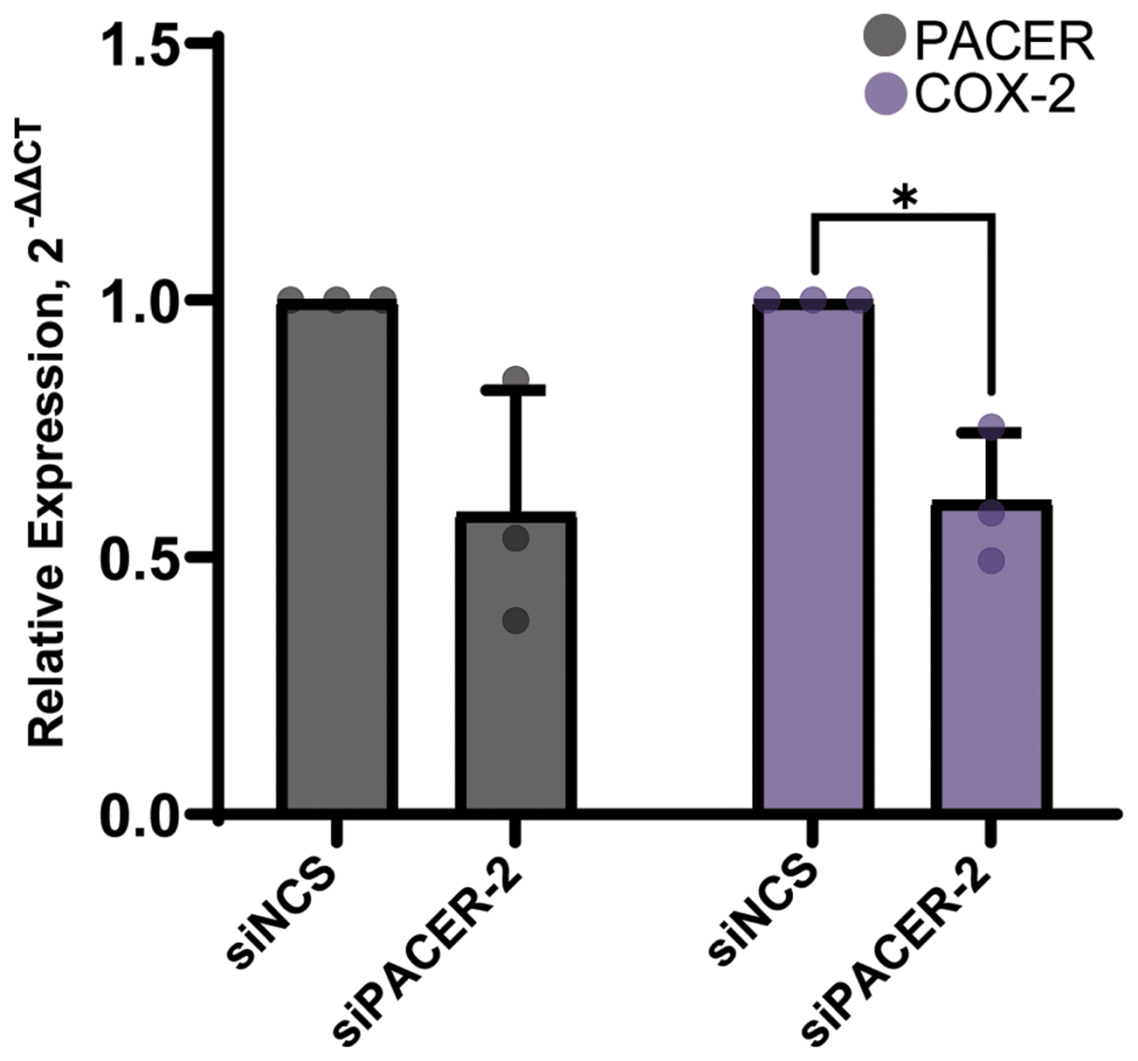
siRNA-mediated knockdown of PACER significantly decreased COX-2 expression. Treatment of A549 cells with a final concentration of 10 nM siRNA duplex decreased PACER expression and significantly decreased the expression of COX-2 (*p* = 0.036) at 24 hours relative to the siNCS control treatment. Error bars indicate standard error of the mean. ^*^ Indicates *p* < 0.05.

To determine if PACER is playing a direct role in the regulation of COX-2 mRNA expression, we performed an siRNA-mediated knockdown of PACER in A549 cells (Figure 4). We treated A549 cells with a PACER-specific siRNA (siPACER) and a noncoding siRNA control sequence (siNCS). We then analyzed the RNA expression of both PACER and COX-2 using qRT-PCR. A549 cells displayed decreased expression of PACER (t(2.00) = 3.00, *p* = 0.095) and had significantly lower COX-2 expression (t(2.00) = 5.00, *p* = 0.036) 24 hours after treatment with a final concentration of 10 nM siPACER duplex relative to the siNCS control treatment. Our analysis shows that treatment with siPACER reduces PACER expression and directly limits PACER’s ability to stimulate transcription of COX-2 mRNA.

### Cytokines stimulate PACER expression in A549 cells

**Figure 5:**
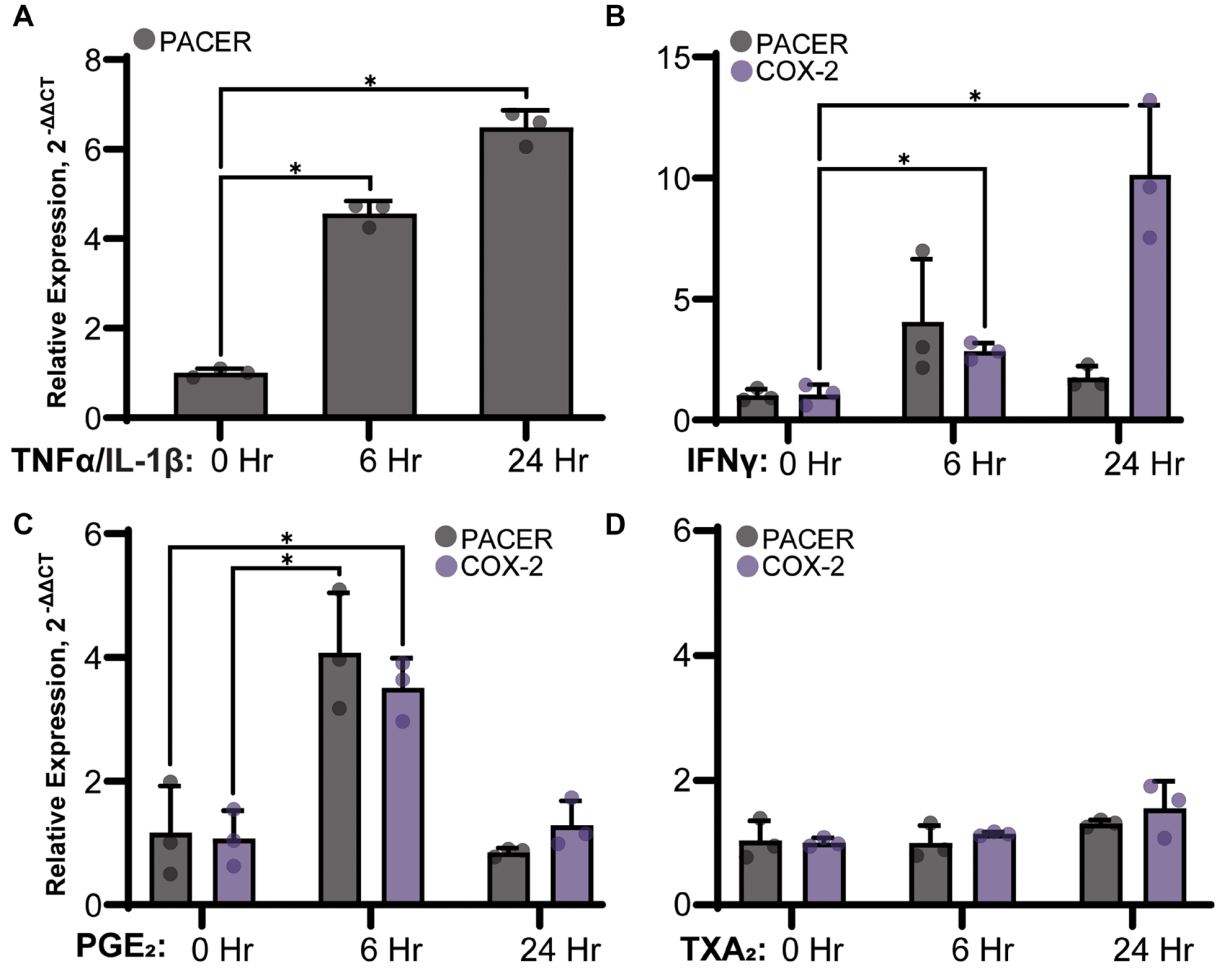
Stimulation of PACER expression in A549 cells treated with (**A**) 50 ng/mL TNFα and 10 ng/mL IL-1β IL-1β, (**B**) 50 ng/mL IFNγ, (**C**) 1 μM PGE_2_ or (**D**) 1 μM TXA2. RNA was isolated from A549 cells 24 hours after treatment and was analyzed for changes in PACER and COX-2 expression relative to the untreated control via qRT-PCR. (A) Cytokine stimulation with TNF-α/IL-1β significantly increased PACER expression in a time dependent manner (6 hours, *p* = 0.001, 24 hours, *p* = 0.001). (B) IFNγ could stimulate expression of COX-2 but not PACER (6 hours, *p* = 0.005, 24 hours *p* = 0.030). (C) PGE_2_ treatment increased both PACER and COX-2 expression at 6 hours (*p* = 0.016, *p* = 0.003). (D) Treatment with TXA2 did not have any significant effect on PACER or COX-2 expression. Error bars indicate standard error of the mean. ^*^ indicates *p* < 0.05.

TNF-α and IL-1β are two major inflammatory cytokines known to stimulate COX-2 expression in A549 cells [45]. We have previously shown that TNF-α and IL-1β treatment stimulates COX-2 protein expression; however, their role in PACER expression has not been explored [46]. To determine the role of cytokine stimulation on PACER expression, we treated A549 cells with a combination of TNF-α (50 ng/mL) and IL-1β (10 ng/mL) for up to 24 hours (Figure 5A). After treatment, we analyzed A549 cell lysates for changes in PACER expression via qRT-PCR. We found TNF-α/ IL-1β treated samples significantly increased PACER expression at the 6-hour (t(2.46) = 21.24, *p* = 0.001) and 24-hour (t(2.24) = 24.21, *p* = 0.001) time points compared with the vehicle control. Additionally, we treated A549 cells with the proinflammatory cytokine IFNγ (Figure 5B). While COX-2 expression significantly increased in response to IFNγ treatment at the 6-hour (t(3.86) = 5.74, *p* = 0.005) and 24-hour (t(2.08) = 5.40, *p* = 0.030) time points, we did not see a statistically significant change in PACER expression.

Our results indicate a distinct difference in PACER expression resulting from stimulation with TNF-α/ IL-1β compared with stimulation with IFNγ. TNF-α/ IL-1β dramatically induced PACER expression, while the effect of IFNγ treatment was less robust. IFNγ is typically thought of as a proinflammatory cytokine; however, some studies indicate IFNγ has a dual role as both a pro and anti-inflammatory signaling molecule [47]. The role of cytokine signaling in the inflammatory response to LUAD is undoubtedly complex and requires further investigation.

### Products of the cyclooxygenase pathway specifically stimulate PACER expression

PGE_2_ is known to have a dual role in regulating COX-2 expression through a complex feedback mechanism [48, 49]. To test if PGE_2_ plays a direct role in regulating PACER expression, we treated A549 cells with a final concentration of 1 μM PGE_2_ (Figure 5C). A549 cells treated with PGE_2_ showed significantly elevated PACER (t(3.784) = 4.120, *p* = 0.016) and COX-2 (t(3.99) = 6.34, *p* = 0.003) expression at 6 hours. At 24 hours, PACER and COX-2 expression returned to near baseline levels. Our results suggest the expression of PACER is transient in nature leading to sustained COX-2 expression. This fits with the current view of PACER as an intermediate step in the transcriptional activation of COX-2.

Next, we wanted to determine if the ability to regulate PACER is unique to PGE_2_ or if other products of the AA pathway can also stimulate PACER expression. We treated A549 cells with TXA_2_ (Figure 5D). PGE_2_ and TXA_2_ are both eicosanoids synthesized from PGH_2_. However, PGE_2_ is primarily synthesized by the synthase mPGES-1, while TXA_2_ is synthesized by thromboxane-A synthase. TXA_2_ has roles in cell growth and proliferation and is known to be dysregulated in lung cancer [50]. Treatment of A549 cells with 1 μM TXA_2_ did not result in any robust change to PACER or COX-2 RNA expression, suggesting that other eicosanoids do not stimulate PACER and that PACER is specifically modulated by PGE_2_ biosynthesis.

### Inhibition of COX-2 activity and COX-2-activating transcription factors

**Figure 6:**
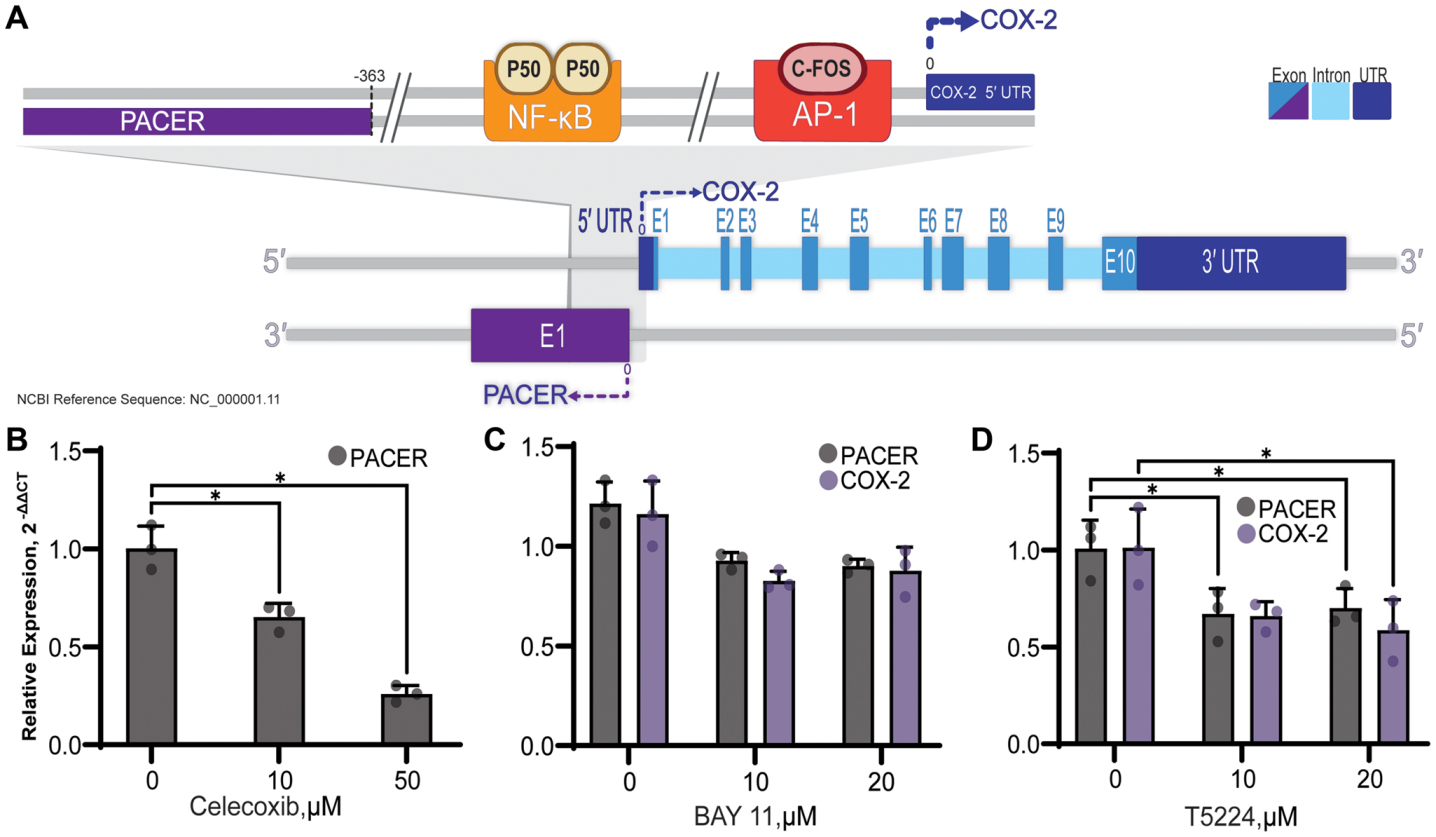
Diagram of PACER and COX-2 genes and promotor. (**A**). The COX-2 gene is comprised of 10 exons, and the 5′ and 3′ untranslated regions are indicated. PACER lies -363bp downstream of the COX-2 transcriptional start site in the antisense orientation and consists of a single exon. NF-κB and AP-1 transcription factor binding sites are shown in their relative position to the COX-2 and PACER transcriptional start sites. The COX-2 promoter contains several other transcription factor binding sites that are not depicted here for clarity. (**B**) Use of specific inhibitors of both COX-2 activity and COX-2 inducing transcription factors can reduce PACER expression. (B) Inhibition of COX-2 in A549 cells with celecoxib decreased expression of PACER at both 10 μM and 50 μM doses (*p* = 0.015, *p* = 0.003). (**C**) BAY 11-7085 (BAY 11) treatment resulted in decreased expression of both PACER and COX-2 at 10 μM and 20 μM doses (*p* = 0.144, *p* = 0.128). (**D**) C-Fos/AP-1 inhibition with T-5224, significantly reduced the expression of PACER at both 10 μM and μM doses (*p* = 0.042, *p* = 0.048). Error bars indicate standard error of the mean. ^*^ indicates *p* < 0.05.

Exogenous PGE_2_ is known to stimulate COX-2 expression in lung cancer cells [51]. As COX-2 expression and PGE_2_ production are both increased in lung cancer, we wanted to determine if high levels of PGE_2_ is stimulating PACER expression. To test this, we treated A549 cells with celecoxib, a specific inhibitor of COX-2 (Figure 6B). Treatment with both 10 μM and 50 μM concentrations significantly lowered PACER expression (t(3.30) = 4.61, *p* = 0.015, t(2.55) = 10.71, *p* = 0.003), suggesting that PACER transcription is in part regulated by feedback from COX-2-mediated production of PGE_2_.

The promoter region of COX-2 overlaps with the antisense start site of PACER transcription and houses binding sites for several transcription factors, including NF-κB and c-Fos/c-Jun. Two NF-κB p50 subunits constitutively bound to the NF-κB promotor results in inhibited COX-2 transcription (Figure 6A). To determine the role of NF-κB in the regulation of PACER, we treated A549 cells with BAY 11, an inhibitor of IKK. BAY 11 prevents nuclear translocation of the activating p65 subunit of NF-κB, effectively blocking transcription of COX-2 [52] (Figure 6C). Treatment with BAY 11 resulted in a decrease in both COX-2 and PACER RNA expression at both 10 μM and 20 μM doses. This result confirms that NF-κB is an important regulator of COX-2 and PACER expression in lung cancer cells.

While regulation of PACER by NF-κB has been shown in CRC and now in lung cancer, induction of PACER expression by other COX-2-related transcription factors has not been investigated. AP-1 is a heterodimeric transcription factor composed of c-Fos and c-Jun and binds to the promoter region of COX-2 approximately 65 bp upstream of the transcription initiation site [53] (Figure 6A). We treated A549 cells with T-5224, an inhibitor of c-Fos/AP-1, to determine if transcription factors other than NF-κB can stimulate PACER expression (Figure 6D). Treatment of A549 cells with 20 μM T-5224 significantly decreased expression of both PACER and COX-2 RNA (t(3.52) = 2.98, *p* = 0.048, t(3.79) = 2.91, *p* = 0.047). Inhibition of PACER expression by T-5224 has not been previously described. Regulation of PACER by T-5224 indicates that additional undescribed mechanisms can alter PACER expression. We are interested in further investigating this regulatory mechanism to determine if inhibition of AP-1 binding is directly regulating PACER transcription.

## DISCUSSION

While the five-year survival rate of lung and bronchial cancer has marginally improved in recent years to 19%, late detection of tumors and limited screening programs result in delayed diagnosis and initial treatment of the disease [54, 55]. In addition, many current therapies do not adequately address the critical role of inflammatory enzymes and their metabolites, which largely regulate the interactions between tumor cells, healthy tissue, and the immune system in the context of cancer. One method of PGE_2_ regulation is the use of non-steroidal anti-inflammatory drugs (NSAIDs) such as celecoxib. Celecoxib is a selective inhibitor of COX-2. Clinical trials targeting patients with advanced NSCLC showed that celecoxib is effective in combination with immune checkpoint inhibitors and targeted therapies but also increases the risk of adverse effects such as hematological toxicity and cardiovascular complications [56]. Additional strategies to regulate COX-2-derived PGE_2_ are essential to optimize and maximize the clinical benefits of non-targeted therapies such as NSAIDs.

Various mechanisms have evolved to fine-tune control of COX-2 expression, implying the critical importance of tightly regulating PGE_2_ production. Transcriptional regulation of COX-2 is mediated through its promoter region by binding of both inhibitory and activating transcription factors. Post-transcriptional control of COX-2 is accomplished through the presence of 3′ AU-rich elements (AREs), as well as other mechanisms such as alternative polyadenylation [33, 35, 57]. Destabilizing proteins such as ARE RNA-binding protein-1 bind to AREs on COX-2 mRNA, inducing rapid deadenylation and mRNA decay [57]. MiRNAs can also regulate PGE_2_ production by altering COX-2 mRNA expression. Previously, we investigated the role of miRNAs miR-708-5p and miR-146a in the regulation of AA signaling. We demonstrated that dysregulation of miR-708-5p and miR-146a leads to significantly increased production of PGE_2_ in lung cancer cells [28, 31, 58]. COX-2 protein degradation is mediated by multiple pathways, providing additional post-translational mechanisms regulating COX-2-derived PGE_2_ production [59].

Our knowledge of lncRNAs is rapidly growing and changing as investigators identify many new and unique roles that these molecules play in human physiology. LncRNAs have a diverse range of functions, including roles in metabolism, transcriptional regulation, differentiation, and immune response [60–62]. The lncRNA PACER provides yet another level of transcriptional regulation by managing the binding of COX-2 transcription factors. Here, we build upon previous research establishing PACER’s role in COX-2 regulation and cancer progression to understand how proinflammatory cytokines can regulate PACER and downstream transcription of COX-2 in lung cancer. Our results demonstrate a clear relationship between PACER expression and the transcriptional regulation of COX-2 in lung cancer cell lines.

Krawczyk et al. first identified and characterized PACER as a regulator of COX-2 expression [41]. They additionally showed that PACER sequesters the p50 subunit of NF-κB, promoting COX-2 expression. Krawczyk et al. discuss the potential clinical benefits of PACER regulation. Qian et al. were the first to investigate the regulation of PACER in cancer by utilizing short hairpin RNA (shRNA) to specifically target PACER overexpression in osteosarcoma [63]. They show that shRNA-mediated knockdown of PACER reduced levels of COX-2 mRNA and protein in osteosarcoma cells in an NF-κB dependent manner. Additionally, Sun et al. showed that siRNA-mediated knockdown of PACER resulted in reduced COX-2 expression indicating that PACER has a direct role in COX-2 transcription proliferation and invasion in CRC cells.

Consistent with Qian et al.’s observation in osteosarcoma cells [63] and Sun et al.’s observations in CRC [42], our initial bioinformatic investigation of PACER in lung cancer revealed a strong correlation between PACER and COX-2 expression (Figure 1C). We found that high PACER and COX-2 expression can predict decreased DSS in females aged 65 years or older (Figure 2C). We stratified this group by disease stage and found high PACER and COX-2 expression negatively impact DSS in patients with stage I or II; however, the small size of the available dataset prevents us from making any statistically significant conclusions (data not shown). Previous studies have indicated sex-based differences in COX-2’s role in inflammatory disease [64]. The recent publication by Sayad et al. observed sex-specific overexpression of PACER in the blood of female patients with periodontitis, an inflammatory gum disease [65]. Both estrogen and testosterone can affect cyclooxygenase expression, but the role of hormones in PACER expression is unexplored. Future *in vitro* studies should be prepared to consider the effect of sex on PACER expression and treatment efficacy.

Interestingly, we did not observe the same trends in LUSC. We found a much weaker correlation between PACER and COX-2 expression in LUSC patients (data not shown). Additionally, we did not find a difference in survival between female patients aged 65 years or older with high PACER and COX-2 expression versus low expression (Supplementary Figure 1C). While the role of PACER in LUSC requires further investigation, there are apparent differences between the expression of PACER in LUAD and LUSC. This should be taken into consideration when developing any treatment strategy targeting the regulation of PACER or COX-2. The size of TCGA patient cohorts limits our bioinformatic analysis. Further investigation of PACER and COX-2 cooperation in clinically-derived samples will help clarify the effect of PACER expression on clinical outcomes.

We chose to conduct the remainder of our experiments using A549 cells because of the observed positive correlation between PACER and COX-2 expression and high levels of both genes relative to their expression levels in Beas2B cells (Figure 3A). In the future, it may be efficacious to examine a model with more moderate PACER and COX-2 expression levels to account for observations that may have occurred from significant overexpression alone. That being said, the high expression of PACER in A549 cells allowed us to detect a broader range of changes in PACER regulation resulting from our experimental treatments.

We wanted to show PACER’s direct role in the regulation of COX-2 in lung cancer cells. Sun et al. previously reported the use of siRNA to mediate the knockdown of PACER expression in CRC cells lines. We used a modified version of the siRNA they originally reported to mediate the knockdown of PACER in A549 cells. We observed a decrease in PACER expression and a significant reduction in COX-2 expression compared to our siNCS control treatment. The observed decrease in COX-2 expression after specific knockdown of PACER demonstrates that PACER RNA alone can alter COX-2 mRNA expression in lung cancer cells.

Previous research has shown that PACER is stimulated by LPS, a strong proinflammatory stimulus [41]. We found that treating A549 cells with proinflammatory cytokines TNF-α/IL-1β stimulates the NF-κB pathway and significantly increases PACER expression (Figure 5A). This is consistent with the idea that PACER may be acting at an intermediate regulatory step in COX-2 transcription in LUAD. While not statistically significant, IFNγ did increase PACER expression at the 6-hour time point, followed by a large increase in COX-2 mRNA expression at the 24-hour mark (Figure 5B). This pattern of expression supports the idea that cytokine-induced PACER transcription precedes the transcription of COX-2, potentially leading to a delayed increase in COX-2 expression. As mentioned previously, the complexity of cytokine signaling networks in tumor tissue is immensely intricate and requires further detailed analysis.

While not significant, we did observe a decrease in PACER expression after inhibition of NF-κB with BAY 11, consistent with Krawczyk et al.’s [41] theory of PACER regulation (Figure 6C). We also observed a decrease in PACER expression in lung cancer cells after treatment with the c-Fos/AP-1 inhibitor T-5224 (Figure 6D). Further investigation of this potential regulatory mechanism will be required in the future to determine if c-Fos/AP-1 is directly involved in PACER transcription or indirectly regulating PACER expression through inhibition of COX-2 and downstream production of PGE_2_.

Eicosanoid-treated A549 cells exhibited increased expression of PACER when treated with PGE_2_, but no significant change was observed after TXA_2_ treatment (Figure 5C and 5D). This specificity allows PGE_2_ to precisely stimulate PACER expression and strengthens the idea of a regulatory feedback loop, allowing PGE_2_ to modulate COX-2 expression through PACER. Because other eicosanoids do not stimulate PACER expression, COX-2 regulation is effectively insulated from the impact of other AA-derived inflammatory processes, allowing PGE_2_ an additional level of control over this cycle. Moreover, inhibition of COX-2 with celecoxib, effectively inhibiting PGE_2_ production, resulted in a significant decrease in the expression of PACER. Given the intimate transcriptional relationship between PACER and COX-2, it is not surprising that such a feedback mechanism would exist (Figure 6B).

**Figure 7:**
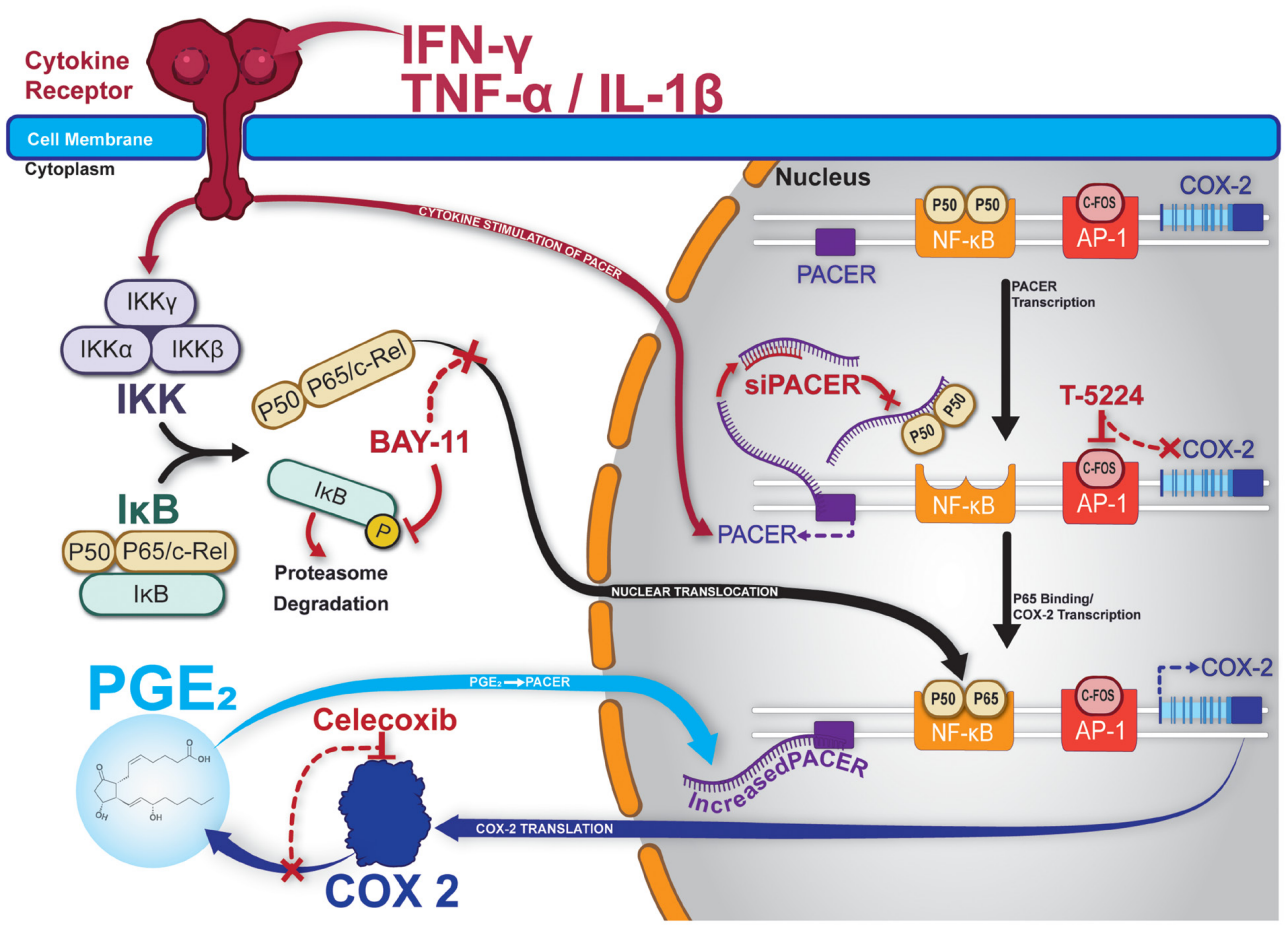
Graphical summary and model of PACER in lung cancer cells. Treatment with cytokines stimulates expression of PACER. Cytokines also mediate the cleavage of NF-κB subunits p50/p65 from IκB. PACER sequesters the P50 homodimer of NF-κB allowing p50/p65 heterodimers to promote transcription of COX-2. Increased COX-2 transcription results in increased production of PGE-2. Increased levels of PGE_2_ further promotes PACER expression. BAY-11 inhibits the phosphorylation of IκB, preventing free p50/p65 and transcriptional may inhibit activation of COX-2. Celecoxib specifically inhibits COX-2 enzymatic activity and downstream production of PGE_2_. T-5224 specifically inhibits c-Fos/AP-1 and transcriptional activation of COX-2. siPACER specifically targets PACER for degradation inhibiting downstream expression of COX-2 mRNA.

We provide a visual outline of our experiments in Figure 7. We illustrate the relationship between cytokine-stimulated PACER production and downstream production of COX-2. We also note the targets of each of the inhibitors used in our characterization of PACER. Overall, our results show that PACER is a key regulatory element in the inflammatory response of LUAD. Our bioinformatic analysis provided evidence of PACER dysregulation in LUAD. Additionally, we identified the correlation and coordinate expression of PACER and COX-2 in specific subsets of adenocarcinoma patients. In this communication, we have characterized a feedback mechanism involving the PGE_2_/PACER/COX-2 axis and show that PACER transcription may be regulated by mechanisms other than NF- κB signaling. We suggest PACER as a potential therapeutic target in LUAD due to its role in COX-2 regulation. Treatments targeting PACER may help increase the efficacy of current COX-2 inhibitors or act as a stand-alone therapy.

## MATERIALS AND METHODS

### Data sources and pre-processing

FPKM-normalized RNA-seq data from all The Cancer Genome Atlas (TCGA) studies were obtained from the NCI Genomic Data Commons (GDC, https://portal.gdc.cancer.gov/) using the GDC RNASeq Tool (https://gdc.cancer.gov/content/gdc-rnaseq-tool). ArrayStudio (OmicSoft/QIAGEN) was used to compare gene expression across all TCGA patient study cohorts. The corresponding clinical data for LUAD and LUSC patients were downloaded from the respective TCGA Pan-Cancer Atlas studies available on cBioPortal [44].

Only tumor samples were considered. In the case of multiple samples per patient, the sample with the highest variation in gene expression was kept. Principal components analysis (PCA) using the *prcomp* function [66] in R was conducted on each dataset to identify possible outliers based on tissue source site [67]. Nine samples in LUAD were found to cluster by site and removed from subsequent analysis. No LUSC samples were removed. The resulting dataset sizes for LUAD and LUSC consisted of 439 and 484 samples, respectively.

### Survival analysis

Patients expressing COX-2 and PACER were classified into “high,” “medium,” or “low” expression groups by dividing the total range of expression values into thirds. The “high,” “medium,” or “low” expression groups were defined by setting cutoffs at the top one-third and bottom one-third of the total COX-2 or PACER expression range. Ranking was performed separately for COX-2 and PACER. Ranked patients were then selected into two groups based on COX-2 and PACER coordinate expression: patients with both “high” COX-2 and PACER expression (falling into the top third of their respective expression distribution), denoted as “COX-2 & PACER high,” and patients with both “low” COX-2 and PACER expression (falling into the bottom third of their respective expression distribution), denoted as “COX-2 & PACER low.”

Kaplan-Meier (KM) survival and COX proportional hazards analyses were conducted between patient groups with “COX-2 & PACER high” and “COX-2 & PACER low” in different stratified clinical subsets (based on gender, age, and tumor stage) using the survfit and coxph function from the survival package in R [68]. The clinical endpoint for survival analysis in both TCGA LUAD and TCGA LUSC cohorts was DSS. The statistical significance of the KM survival curves was assessed by log-rank test, and the statistical significance of the hazard’s ratio was assessed using the Wald test and adjusted for clinical covariates (gender, age, tumor, stage, when stratification on these variables was not done).

### Mammalian cell culture

Beas2B and A549 cells (ATCC) were grown in Dulbecco’s Modified Eagle’s Medium (DMEM, Sigma-Aldrich). H1299, H1975, H1373, and H23 cells were grown in Roswell Park Memorial Institute-1640 Medium (RPMI, Sigma-Aldrich). Media were supplemented with 10% FBS (Atlanta Biologicals), 4 mM L-glutamine (Thermo Fisher Scientific), and 1% Penicillin/Streptomycin (Corning). All cells were incubated at 37°C in a 5% CO_2_ incubator. Please note that the strain of Beas2B cells used here does not form tumors in nude mice [69].

### RNA isolation and quantitative reverse transcriptase-PCR

Total RNA was isolated from cells using TRIzol Reagent (Thermo Fisher Scientific) following the manufacturer’s suggested protocol. For both PACER and COX-2 RNA analysis, complementary DNA (cDNA) was generated using Maloney Murine Leukemia Virus Reverse Transcriptase (M-MuLV RT, New England Biolabs) following the standard protocol. Each cDNA preparation contained 1 μg of total RNA.

Quantitative reverse transcriptase-PCR (qRT-PCR) was performed using a Bio-Rad CFX96 Real-Time C1000 Touch Thermal Cycler and the following cycling conditions: (1) 94°C for 3 min, (2) 40 cycles of 94°C for 15 sec, 55°C for 30 sec, 68°C for 30 sec (collection step). The following primers were used to detect and quantify RNA expression: PACER forward 5′-TGTAAATAGTTAATGTGAGCTCCACG-3′, PACER reverse 5′-GCAAATTCTGGCCATCGC-3′ (Millipore Sigma), GAPDH forward 5′-CCACCCATGGCAAATTCCATGGCA-3′, GAPDH reverse 5′-TCTAGACGGCAGGTCAGGTCCACC-3′ (Integrated DNA Technologies), COX-2 QuantiTect Primer Assay (NM_000963, Qiagen). Amplification was performed using SYBR green master mix containing Hot Start Taq DNA polymerase (New England Biolabs). No template controls and cDNA preps containing no reverse transcriptase were included to ensure samples were not contaminated. For additional validation, melt curve analysis and electrophoresis of amplified products were performed. Log base 2 negative quantitative comparative CT (2^−ΔΔCT^) analysis was used to quantify gene expression changes relative to GAPDH.

### Cytokine and eicosanoid treatments

A549 cells were seeded in individual 12-well plates at a density of 1 × 10^5^ cells/well and were allowed to grow overnight. The following day, cells were washed with 1X PBS and incubated overnight in serum-free medium (DMEM base medium, 4 mM L-glutamine, and 1% Penicillin/Streptomycin) to promote synchronization. The next morning, cells were treated with 50 ng/mL IFNγ (PeproTech) or a combination of 50 ng/mL TNF-α and 10 ng/mL IL-1β (PeproTech) in serum-free medium for the indicated time points, then RNA was isolated. A549 cells were seeded as described above for treatments with PGE_2_ and carbocyclic thromboxane A2 (TXA_2_). The following morning after seeding, cells were treated with 1 μM PGE_2_ (Sigma-Aldrich) or TXA_2_ (Cayman Chemical) in complete medium for the indicated time points. RNA was then isolated as described above.

### Inhibitor treatments

A549 cells were seeded in individual 12-well plates at a density of 1 × 10^5^ cells/well and were allowed to grow overnight. The next morning celebrex, BAY 11-7085 (BAY 11), or T-5224 were administered in complete medium at the indicated concentrations; Celecoxib (Cayman Chemical) was used to inhibit COX-2 activity, T-5224 (Cayman Chemical) was used to inhibit AP-1 binding to DNA, and BAY 11 (Cayman Chemical) was used to inhibit NF-κB activation. DMSO alone was used as the control treatment. RNA was isolated 6–24 hours after treatment.

### siRNA treatments

A549 cells were seeded in 6-well plates at a density of 2.0 × 10^5^ cells/well in antibiotic-free DMEM supplemented with 10% FBS and 4 mM L-glutamine. 24 hours after treatment, siRNA duplex and Lipofectamine RNAiMAX transfection reagent (Invitrogen) were separately added to serum-free DMEM, then gently mixed and incubated together for approximately 15 minutes. Cells were then transfected with PACER siRNA (siPACER) or a noncoding siRNA control sequence (siNCS) at a final concentration of 10 nM. The following siRNA sequences originally reported by Sun et al. [42] were slightly modified and synthesized by Integrated DNA Technologies: siPACER sense, 5′-CAUAGGAGAUACUGGUAAAUU-3′ and antisense, 5′-UUUACCAGUAUCUCCUAUGUU-3′; siNCS sense, 5′-UUCUCCGAACGUGUCACGUUU-3′ and antisense, 5′-ACGUGACACGUUCGGAGAAUU-3′. Cells were lysed 24 hours after transfection, and RNA was isolated for qRT-PCR analysis as described above.

### Statistical analysis

All statistical analyses of cell-based assays were performed using GraphPad Prism (version 9.2) (GraphPad Software). qRT-PCR data represent the average of at least three independent biological replicates. Each biological replicate was measured with *n* ≥ 2 technical replicates per target gene per independent experiment. Two-tailed Welch’s *t*-test was performed to compare expression levels of PACER and COX-2 in the treatment conditions to respective control groups for all cell-based assays. For all tests, *p* < 0.05 is considered statistically significant.

## SUPPLEMENTARY MATERIALS



## Abbreviations

COX-2: cyclooxygenase 2
PACER: p50-associated COX-2 extragenic RNA
lncRNA: long non-coding RNA
AA: arachidonic acid
LUAD: lung adenocarcinoma
LUSC: lung squamous cell carcinoma
