# Supplementary material for: PACER lncRNA regulates COX-2 expression in lung cancer cells
Source: Oncotarget. 2022 Feb 4;13:291–306. doi: 10.18632/oncotarget.28190 (PMC8815784; doi:10.18632/oncotarget.28190)
Supplement: Supplementary file 1 [file oncotarget-13-28190-s001.pdf]

# PACER lncRNA regulates COX-2 expression in lung cancer cells

## SUPPLEMENTARY MATERIALS

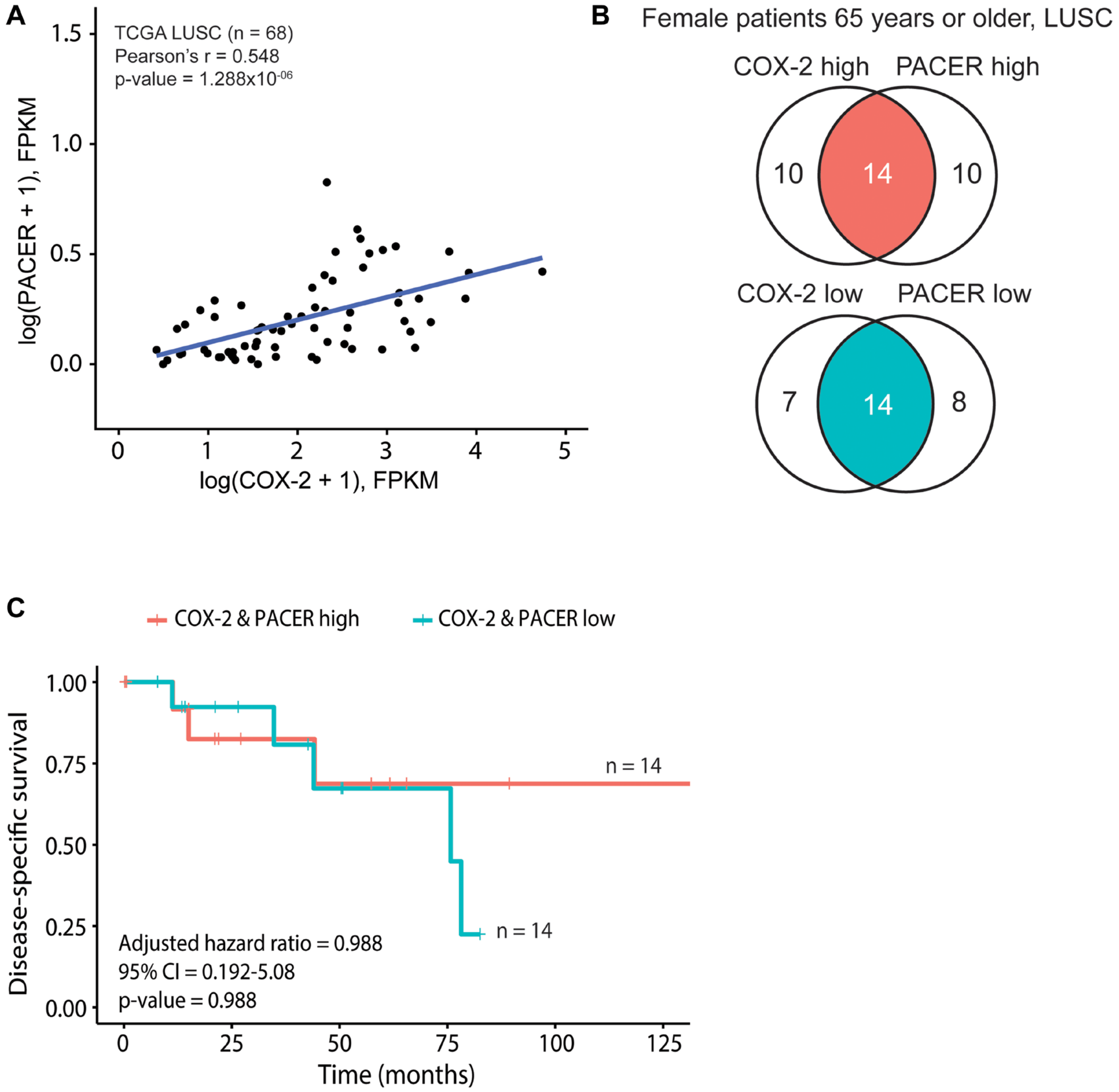


**Supplementary Figure 1: COX-2 and PACER coordinate expression do not contribute to survival differences in a subset of older female patients in lung squamous cell carcinoma (LUSC).** Panels (**A**–**C**) show analyses conducted on The Cancer Genome Atlas (TCGA) LUSC datasets. (A) Scatterplot of COX-2 and PACER expression, with a fitted linear regression line. The Pearson's correlation coefficient (r) and corresponding *p*-value calculated from the logged data are shown. (B) Venn diagrams depicting the identification of "COX-2 & PACER high" and "COX-2 & PACER low" groups in female patients aged 65 years or older. (C) Kaplan-Meier survival curves between the high and low patient groups defined by B, shown with the corresponding hazard ratio, confidence interval (CI), and Wald *p*-value calculated from a Cox proportional hazards model adjusted for tumor stage.
